# Oestrogen deprivation induces chemokine production and immune cell recruitment in in vitro and in vivo models of oestrogen receptor-positive breast cancer

**DOI:** 10.1186/s13058-021-01472-1

**Published:** 2021-10-03

**Authors:** Jody Hazlett, Virginia Niemi, Aziz Aiderus, Katelyn Powell, Lyn Wise, Roslyn Kemp, Anita K. Dunbier

**Affiliations:** 1 Department of Biochemistry, University of Otago, Dunedin, New Zealand; 2 Department of Microbiology and Immunology, University of Otago, Dunedin, New Zealand; 3 Department of Pharmacology and Toxicology, University of Otago, Dunedin, New Zealand

**Keywords:** Non-steroidal anti-inflammatory drugs, Oestrogen receptor, Breast cancer, Endocrine therapy

## Abstract

**Background:**

Oestrogen receptor-positive (ER+) breast cancer is commonly treated using endocrine therapies such as aromatase inhibitors which block synthesis of oestradiol, but the influence of this therapy on the immune composition of breast tumours has not been fully explored. Previous findings suggest that tumour infiltrating lymphocytes and immune-related gene expression may be altered by treatment with aromatase inhibitors. However, whether these changes are a direct result of impacts on the host immune system or mediated through tumour cells is not known. We aimed to investigate the effect of oestrogen deprivation on the expression of chemokines and immune infiltration in vitro and in an ER+ immunocompetent mouse model.

**Methods:**

RT-qPCR and a bead-based Bioplex system were used to investigate the expression of chemokines in MCF-7 breast cancer cells deprived of oestrogen. A migration assay and flow cytometry were used to measure the migration of human peripheral blood mononuclear cells (PBMCs) to MCF-7 cells grown without the main biologically active oestrogen, oestradiol. Using flow cytometry and immunohistochemistry, we examined the immune cell infiltrate into tumours created by injecting SSM3 ER+ breast cancer cells into wild-type, immunocompetent 129/SvEv mice.

**Results:**

This study demonstrates that oestrogen deprivation increases breast cancer secretion of TNF, CCL5, IL-6, IL-8, and CCL22 and alters total human peripheral blood mononuclear cell migration in an in vitro assay. Oestrogen deprivation of breast cancer cells increases migration of CD4+ T cells and decreases migration of CD11c+ and CD14+ PBMC towards cancer cells. PBMC migration towards breast cancer cells can be reduced by treatment with the non-steroidal anti-inflammatory drugs, aspirin and celecoxib. Treatment with endocrine therapy using the aromatase inhibitor letrozole increases CD4+ T cell infiltration into ER+ breast cancer tumours in immune competent mice.

**Conclusions:**

These results suggest that anti-oestrogen treatment of ER+ breast cancer cells can alter cytokine production and immune cells in the area surrounding the cancer cells. These findings may have implications for the combination and timing of anti-oestrogen therapies with other therapies.

**Supplementary Information:**

The online version contains supplementary material available at 10.1186/s13058-021-01472-1.

## Introduction/background

Breast cancer is the most commonly diagnosed cancer amongst women worldwide [1] and oestrogen receptor-positive (ER +) breast cancer accounts for approximately 80% of breast cancer cases [2, 3]. Aromatase inhibitors (AIs) block synthesis of oestradiol, the main biologically active oestrogen and ligand for the ER, and are frequently used in treatment of ER+ breast cancer in postmenopausal women [4]. Treatment with AIs such as anastrozole and letrozole, or culturing ER+ cells in vitro in media deprived of oestrogen reduces tumour cell proliferation [5, 6]. However, clinical benefit rates are only 50–75% in the neoadjuvant setting and may be lower in advanced disease [6–9]. The risk of recurrence is highest during the first five years after diagnosis [10], but remains at 10–41%, depending on the tumour and nodal status [11]. Consequently, there is significant need for improved therapies and understanding of the mechanisms through which ER+ cancers resist treatment with AIs.

Accumulating evidence suggests that oestrogen affects immune responses at both a systemic and tissue-specific level. Differences in the rates of autoimmune diseases [12], CD4+ T cell counts [13] and antibody responses [14] between males and females are likely to be driven at least in part by sex hormones [15]. Oestrogen receptors are broadly expressed in many cell types involved in the immune response [16]. Recently, infiltrating immune cells have been shown to increase in tumours from patients treated with AIs, compared to pre-treatment levels of the same patients [17].

In the pre-surgical setting, lymphocytic infiltration in breast tumours and expression of immune-related genes are associated with poorer anti-proliferative response to AI treatment in ER+ tumours [18–20], suggesting a link between immune infiltration and response to AI treatment in breast cancer patients. The mechanism(s) by which immune cells are recruited to breast tumours and how this recruitment is affected by AIs is unknown. Chemokine-driven chemotaxis represents a key mechanism through which immune cells can be recruited to tissues [21]. Chemokines are chemotactic cytokines that control the migration and positioning of immune cells in tissues and are critical for the function of the innate immune system [22]. Chemokines can also play many roles in cancer progression, including promotion of angiogenesis, tumour proliferation and metastasis [23, 24].

In this study, we used in vitro and in vivo models of AI treatment alongside analysis of gene expression data from patients treated with AIs. We demonstrate that oestrogen-deprived breast cancer cells produce elevated levels of chemokines compared to non-deprived cells and that this differential expression of chemokines leads to enhanced immune cell migration in vitro. Effects of aromatase inhibition on immune cell composition were also observed in tumours from an allogenic ER+ breast cancer mouse model treated with letrozole.

## Methods

### Analysis of gene expression data

Pre- and on-treatment (14 day) core biopsies were available from postmenopausal women recruited into the anastrozole (1 mg/day)-only arm of a multicenter, randomised, double-blind, phase II neoadjuvant trial of anastrozole alone or with gefitinib in early breast cancer [25]. Sample acquisition, storage, and RNA extraction were conducted as previously described [18]. Following exclusions, paired samples were available from 81 patients. Gene expression profiles were generated on the Illumina HumanWG-6 v2 Expression BeadChip. Illumina raw data were extracted using the BeadStudio software and were transformed and normalised using variance-stabilising transformation and robust spline normalisation method in the Lumi package in R [26]. Gene expression data from this study were deposited by the authors of reference [18] and are available at [27]. Genes differentially expressed between baseline and 2-week samples were identified using a multivariate permutation test implemented in BRB-Array Tools [28]. Immune response genes were identified using GATHER analysis [29].

### Cell culture

MCF-7 or SKBR3 breast cancer cells (obtained from ATCC) were cultured for three days in phenol-free RPMI media supplemented with 10% dextran coated charcoal (both from Sigma, North Ryde, NSW, Australia) stripped bovine serum (DCC) (Gibco, Carlsbad, CA, USA) [5]. Cells were then seeded in triplicate for each sample into six well plates (Greiner Bio-One, Monroe, NC, USA) in hormone-depleted medium with or without 1 × 10^–9^ M β-oestradiol (Sigma, North Ryde, NSW, Australia). Cell lines were shown to be free of mycoplasma by routine testing, and the identity of cell lines was confirmed by STR genotyping.

### RT-qPCR

Total RNA was extracted at specified time points using the Quick-RNA MiniPrep Kit (Zymo Research, Irvine, CA, USA), and 500 ng of RNA of each sample was reverse-transcribed in 10 µL total volume using the PrimeScript™ RT reagent kit (Takara Bio, Mountain View, CA, USA) following the manufacturer’s protocols. RNA was assessed for quality and concentration using a NanoDrop (Thermo Fisher Scientific, Waltham, MA, USA). RT-qPCR was performed using TaqMan Gene expression assays (Applied Biosystems or PrimeTime qPCR Assay, IDT, Coralville, IA, USA) for three chemokines, *CCL5* (Hs.PT.58.40305992), *CCL22* (Custom probe from IDT, sequence 5′/56-FAM/TGCGCGTGG/ZEN/TGAAACACTTCTACT/3IABkFQ/-3′) and *CXCL16* (Hs.PT.58.28311933.gs), as well as three reference genes, *FKBP15* (Hs.PT.49a.2552313), *PUM1* (Hs.PT.56a.1997572) and *TBP* (Hs00427620_m1). RT-qPCR was run on an ABI PRISM 7900 Real-Time PCR System (Applied Biosystems, Foster City, CA, USA). The standard curve method was used for analysis. All determinations were performed in triplicate, and the expression levels for each gene were normalised with respect to the geometric mean of the endogenous reference genes.

### Cytokine assay

MCF-7 or SKBR3 cells were cultured for three days in RPMI media with hormone-depleted bovine serum as described above. 80,000 cells were then seeded into 6 well plates in hormone-depleted medium with (+ E2) or without (− E2). 1 × 10^−9^ M oestradiol and cell supernatant were collected at indicated time points and stored at − 80 °C. Supernatant was concentrated with Microcon YM-3 columns (Millipore, Burlington, MA, USA) before cytokine concentrations were simultaneously measured using a selection of 12 analytes (Flt-3 ligand, CCL2, CCL3, CCL4, CCL5, CCL7, CCL22, IL-1, IL-6, IL-8, IL-10 and TNF) from the Milliplex Human Cytokine/Chemokine panel (HCYTOMAG-60 K, Millipore, Burlington, MA, USA). The assay was carried out according to manufacturer’s instructions with the following modification of 50 µL of sample loaded instead of 25 µl. Samples were run in triplicate and protein levels determined using the Bio-Plex Luminex System (Bio-Rad, Hercules, CA, USA). The arrays were quantified using Bio-Plex Manager V 4.0 (Bio-Rad, Hercules, CA, USA). Raw data were normalised by dividing the chemokine measurement by the cell number in each well to account for the differences in growth between the two treatments.

### Transwell chemotaxis assay

Chemotaxis assays were performed with 24-well Transwell™ supports (Corning, New York, USA) with 8-μm pore membranes. Briefly, MCF-7 cells were grown with or without 1 × 10^−9^ M oestradiol, such that both treatments resulted in equal cell numbers at 72 h. Human peripheral blood mononuclear cells (PBMCs) were isolated from fresh blood using a Ficoll-Paque (GE Healthcare, Parramatta, NSW, Australia) gradient. Ethical approval for blood collection was obtained from the Human Ethics Committee of the University of Otago. Blood donors were healthy and gave informed consent according to the University of Otago Human Ethics guidelines. 1 × 10^6^ PBMCs were added to the top chamber of the transwell in a total volume of 100 µL. In the bottom chamber, MCF-7 cells were cultured in RPMI media with DCC serum with or without oestradiol. RPMI media with DCC serum were used as a negative control. Phytohemagglutinin (PHA)-conditioned media, used as a positive control to induce cell migration, were prepared from PBMCs stimulated with 50 µg/ml gentamicin, 50 µM hydroxyurea (both from Sigma, North Ryde, NSW, Australia), 3 mM lithium chloride (Scharlau, Barcelona, Spain) and 2.5 µg/ml phytohemagglutinin P (Gibco, Carlsbad, CA, USA) in a total volume of 600 µL [30]. After 4-h incubation at 37 °C, transmigrated PBMCs were counted with a hemocytometer, incubated with specific antibodies (Table 1) and analysed by flow cytometry. For the blocking assay, parapoxvirus chemokine binding proteins PVNZ 112.3 and 112.6 [31] were added to the media in the lower chamber of the transwell assay at the indicated concentrations.

**Table 1:** Panel for flow cytometry

Antigen	Human PBMC		Mouse tumours	
	Fluorophore	Laser channel	Fluorophore	Laser channel
CD3	APC Cy 7	R780/60	FITC	B530/30
CD4	PacBlue	V450/50	APC + H7	R780/60
CD8	V 500	V525/50	APC	R670/14
CD11b	n/a	n/a	AF700	R730/45
CD11c	APC	R670/14	BV421	V450/50
CD14	FITC	B530/30	n/a	n/a
CD19	PE	YG586/15	BB700	B710/50
CD25	n/a	n/a	BV510	V525/50
CD45	PerCP/Cy5.5	B710/50	BV605	V610/20
CD127	n/a	n/a	PE	YG586/15
F4/80	n/a	n/a	PECy5	YG670/30
L/D	Texas red	Y610/20	Texas red	Y610/20

### Orthotopic breast cancer tumour model

All animal experiments were approved by the Animal Ethics Committee of the University of Otago. Adult female 129/SvEv wild-type mice were purchased from Animal Resources Centre (Perth, Australia). Animals were fed an oestrogen-free diet for the duration of the study (Speciality Feeds, Perth, Australia). Mice were anaesthetised with a ketamine/domitor/atropine combination and the area sterilised before 5 × 10^5^ SSM3 cells were injected into the 4th inguinal mammary gland. When the tumour reached approximately 250 mm^3^, mice received either an ovariectomy or sham operation, and the tumour was biopsied with an 18G TruCore II Biopsy instrument (Argon Medical, Frisco, TX, USA). Daily treatment commenced on the same day for a total of 25 days. Letrozole (Sigma, North Ryde, Australia, 1 mg/kg) or the control (5% ethanol) was administered subcutaneously. Tumours were measured daily at two perpendicular diameters and the area calculated as V = (W^2^ x L)/2. Ulceration of tumours required early euthanasia of 20% of the mice at various time points for humane reasons. These mice were excluded from the analysis. At the end of the experiment, the mice were killed, tumours collected and used for either immunohistochemistry (IHC) or fluorescence activated cell sorting (FACS).

### IHC

A subset (Additional file 1, n = 25) of the tumour biopsies were preserved in 10% neutral buffered formalin (NBF) for 24 h, paraffin embedded, and sections cut at 4 µm. Sections were stained with the following antibodies, ERα (1:300 dilution, ab3575) and CD3 (1:150 dilution, ab5690) (Abcam, Cambridge, UK). For CD3 evaluation, areas of stroma were identified and as many 40X fields of view were examined as possible (range 3–7 fields of view, average of 4.33). The number of CD3+ cells in each 40X field of view were counted and the average number calculated for each tumour. To determine the ER status of the tumours, the total number of unstained and stained nuclei was counted, and amount of ER staining expressed as a percentage. Samples were considered ER+ if > 1% of tumour cells showed positive nuclear staining.

### Tumour processing

To examine the SSM3 tumour immune infiltrate, a subset (Additional file 1, n = 15) of tumours was used. A small biopsy was taken from the tumour for IHC, and the tumours were transferred into petri dishes with 7 mL of RPMI and disassociated between two pieces of gauze using a 1-mL syringe. Tumour cells were resuspended in 3 mL of FACS buffer (PBS, 0.5% FCS, 0.01% sodium azide, Prolab, Geldenaaksebaan, Germany) through a 70-μm filter. Cells were frozen at – 80 °C in freezing media (90% FCS and 10% DMSO) for at least 24 h before being transferred to liquid nitrogen. Cells were defrosted in warm RPMI media before use in flow cytometry experiments.

### Flow cytometry

For the PBMC migration assay, migrated cells were collected from the bottom of the 24-well plate and incubated with the following anti-human antibodies; CD3, CD4, CD8, CD11c, CD14, CD45 (Table 1; BioLegend, San Diego, CA, USA) and Texas Red live/dead fixable cell stain (Life Technologies, Carlsbad, CA, USA) at optimised concentrations for 30 min on ice in the dark. The gating strategy is shown in Additional file 2. For the mouse tumour analysis, cells were incubated with the following anti-mouse antibodies; CD3, CD4, CD8, CD11c, CD11b, CD19, CD25, CD45, CD127 and F4/80 (Table 1; BioLegend, San Diego, CA, USA) and Texas Red live/dead fixable cell stain (Life Technologies, Carlsbad, CA, USA) at optimised concentrations for 30 min on ice in the dark. The percent of CD3+ (Additional file 3F, CD3+ CD19- gate) and CD19+ (Additional file 3F, CD3- CD19+ gate) cells was calculated as a percent of Live/Dead CD45+ single cells and the percent of CD4+ cells (Additional file 3E, CD4+ gate) calculated as a percent of Live/Dead CD45+ single CD3+ cells.

Antibody concentrations were determined by titration using either healthy human PBMCs or mouse spleen cells. Fluorescent minus one (FMO) controls were used for all experiments. Samples were resuspended in 300 μL of 1% paraformaldehyde (Sigma-Aldrich, St Louis, MO, USA) and incubated for 30 min, washed and resuspended in FACS buffer and stored at 4 °C for no longer than 3 days. One Comp eBeads (eBiosciences, San Diego, CA, USA) were used for compensation. Acquisition of events was performed on a LSR-FORTESSA using FacsDIVA (version 8.0, BD). Data were exported as Flow cytometry standard 3.1 files and analysed using FlowJo (version 10.0.7, Tree Star, Ashland, OR, USA) software.

### Statistical analysis

Statistics were calculated using GraphPad Prism Software (San Diego, CA, USA). At least three independent experiments were performed, and statistical significance between groups was determined by unpaired *t*-tests. *p* ≤ 0.05 was considered statistically significant.

## Results

### Investigation of potential mediators of immune migration in aromatase inhibitor-treated tumours

To identify potential mediators of immune cell recruitment in ER+ breast tumours, we analysed data from 81 patients treated with AIs in a neoadjuvant study [18, 25]. Eighteen genes linked to immune response were identified with a false discovery rate of less than 0.05 and a fold change of greater than 1.5 between baseline and 2 weeks after treatment (Table 2). Interestingly, several genes encoding chemokines were identified as being upregulated in response to AI treatment. These genes included C–C chemokine ligand 5 (*CCL5*) and C–C chemokine ligand 22 (*CCL22*) which have been shown to attract suppressive immune cells to tumours [32–34]. This result led us to hypothesise that withdrawal of oestradiol may stimulate chemokine production which could in turn lead to immune cell recruitment. However, the tumour microenvironment of human tumours is complex and, as this analysis did not reveal which cells produced these chemokine transcripts, we proceeded with further investigation in cultured tumour cells.

**Table 2:** Immune response genes differentially expressed between pre-treatment and 2 weeks samples from 81 patients treated with anastrozole (18) with a false discovery rate of less than 0.05 and a fold change of greater than 1.5 which included “immune response” as a Gene Ontology category

Genbank accession number	Gene symbol	Gene name	FDR	Fold change
NM_032962	*CCL14*	Chemokine (C–C motif) ligand 14	2.89E−05	1.84
NM_006288	*THY1*	Thy-1 cell surface antigen	0.00161	1.73
NM_000609	*CXCL12*	Chemokine (C-X-C motif) ligand 12	0.000507	1.71
NM_001251	*CD68*	CD68 antigen	0.001068	1.71
NM_002990	*CCL22*	Chemokine (C–C motif) ligand 22	0.009704	1.69
NM_001623	*AIF1*	Allograft inflammatory factor 1 (AIF1)	0.00178	1.67
NM_001025159	*CD74*	CD74 antigen	0.000583	1.65
NM_004271	*LY86*	Lymphocyte antigen 86	0.000734	1.65
NM_002123	*HLA-DQB1*	MHC, class II, DQ beta 1	0.001781	1.65
NM_001290	*LDB2*	LIM domain binding 2	0.000211	1.63
NM_005516	*HLA-E*	MHC, class I, E	0.001244	1.63
NM_004512	*IL11RA*	Interleukin 11 receptor, alpha	0.001573	1.63
NM_002985	*CCL5*	Chemokine (C–C motif) ligand 5	0.023538	1.61
NM_033554	*HLA-DPA1*	MHC, class II, DP alpha 1	0.008347	1.61
NM_022555	*HLA-DRB3*	MHC, class II, DR beta 3	0.008369	1.61
NM_000616	*CD4*	CD4 antigen (p55)	0.001385	1.59
NM_001242	*TNFRSF7*	Tumour necrosis factor receptor superfamily 7	0.01003	1.59
NM_022059	*CXCL16*	Chemokine (C-X-C motif) ligand 16	0.008121	1.56

### Oestrogen deprivation upregulates chemokine secretion in MCF-7 human breast cancer cells.

**Fig. 1:**
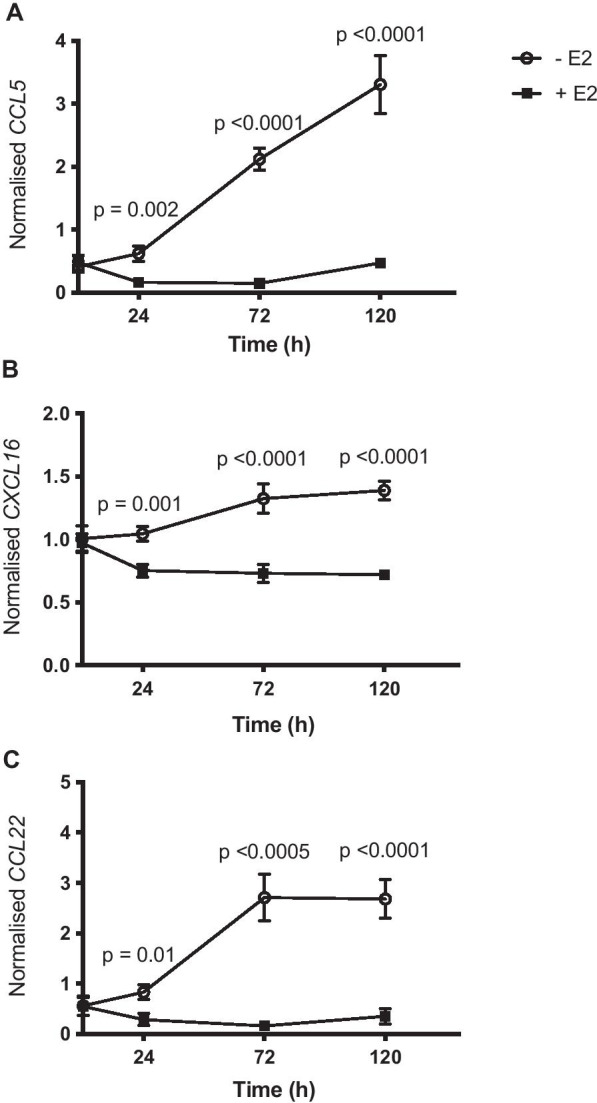
Oestrogen deprivation upregulates expression of chemokines **a** *CCL5*, **b** *CXCL16* and **c** *CCL22* in MCF-7 cells. MCF-7 cells were cultured with (+E2) or without (− E2) 1 × 10^−9^ M oestradiol, and mRNA expression of three chemokines was determined by RT-qPCR at 0, 24, 72 and 120 h. Data are normalised to the mean of three reference genes, *FKBP15*, *TBP* and *PUM1*. Error bars are SEM based on nine biological replicates performed across three independent experiments. *P*-values were calculated using unpaired *t*-tests

To assess the effect of oestrogen deprivation on chemokine gene expression in ER+ MCF-7 breast cancer cells, we used RT-qPCR to measure the mRNA expression of chemokines *CCL5*, *CCL22*, and *CXCL16* from nine biological replicates from three independent experiments*.* mRNA levels of all three chemokines increased in oestrogen-deprived cells relative to cells treated with physiological levels of oestradiol. *CCL5, CXCL16* and *CCL22* were significantly higher (*p* = 0.002, *p* = 0.001 and *p* = 0.01, respectively) in oestrogen-deprived cells 24 h after seeding, with a fold change of 3.73, 1.39 and 2.89, respectively (Fig. 1). At 72 h, all three chemokines showed significant upregulation (fold changes of *CCL5* = 14.4, *CCL22* = 16.9, *p* < 0.0001 for both, and *CXCL16* = 1.8, *p* < 0.0005) which was sustained until 120 h (fold changes of *CCL5* = 7.0, *CCL22* = 7.6 and *CXCL16* = 1.9, *p* < 0.0001 for all) in oestrogen-deprived cells compared to cells supplemented with oestradiol. No significant changes in chemokine gene expression were observed in ER-SKBR3 oestrogen-deprived cells (Additional file 4).

**Fig. 2:**
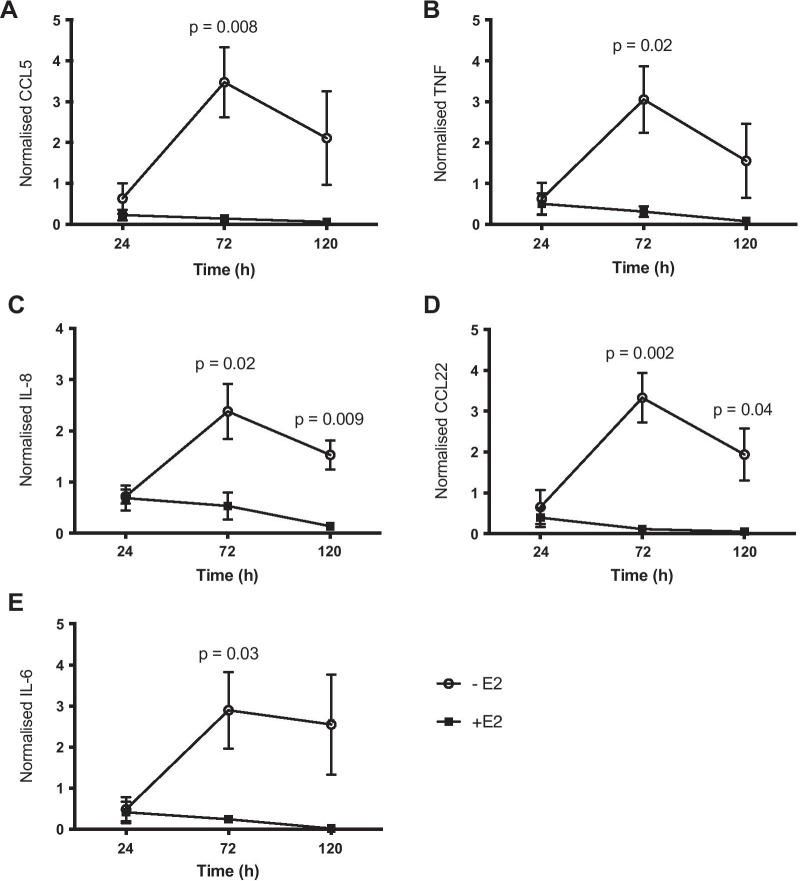
Oestrogen deprivation upregulates cytokine secretion in MCF-7 human breast cancer cells. **a** CCL5, **b** TNF, **c** IL-18, **d** CCL22 and **e** IL-6. MCF-7 cells were cultured with (+E2) or without (− E2) 1 × 10^−9^ M oestradiol, and cell supernatant was collected at 0, 24, 72 and 120 h and analysed with a multiplex assay. Data are normalised to the number of cells in each well. Error bars are SEM based on nine biological replicates performed across three independent experiments. *P*-values were calculated using unpaired *t*-tests

To investigate whether oestrogen deprivation also induces changes in secreted protein levels, a multiplex chemokine/cytokine immunoassay was used to assess protein levels in the media of MCF-7 cells cultured in the presence and absence of oestradiol. Levels of several cytokines were significantly higher in oestradiol-deprived cells (Fig. 2) compared to cells receiving oestradiol at 72 h (CCL5 *p* = 0.008, IL-6 *p* = 0.03, IL-8 *p* = 0.02, TNF *p* = 0.02 and CCL22 *p* = 0.002) and at 120 h (IL-8 *p* = 0.009 and CCL22 *p* = 0.04). The greatest fold change was CCL22 (30.3), followed by CCL5 (24.3). CXCL16 was not available in the multiplex assay. ER-SKBR3 cells did not show consistent increases in levels of the analysed proteins in response to oestrogen deprivation (Additional file 5).

### Oestrogen deprivation of MCF-7 cells alters PBMC migration in vitro

**Fig. 3:**
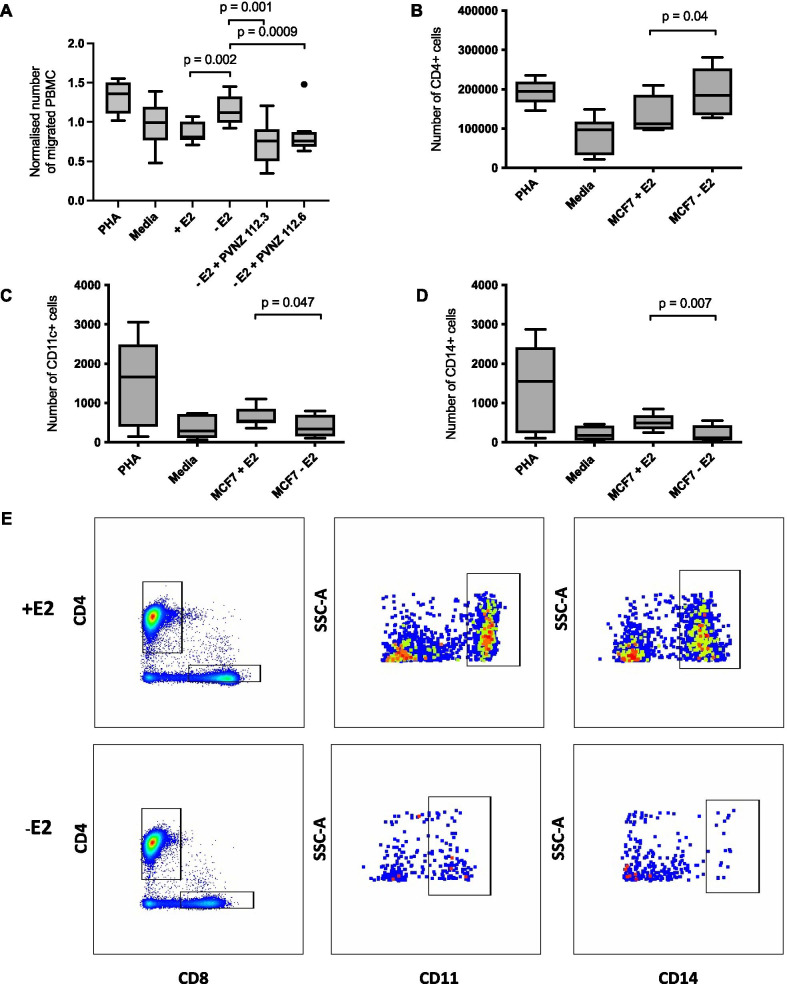
Oestrogen deprivation of MCF-7 cells alters PBMC migration in vitro*.* **a** A transwell assay was used to measure migration of PBMCs towards oestrogen-deprived MCF-7 cells treated CXC-chemokine binding protein (PVNZ 112.3) or CC-chemokine binding protein (PVNZ 112.6). Migration towards oestrogen supplemented (+E2) MCF-7 cells and control media with MCF-7 cells (− E2) was also measured. Data were normalised by dividing values from each experiment by the mean of that experiment. PHA = conditioned media from PBMCs treated with phytohaemagglutinin, a T-cell mitogen. Media = complete media only. Cells from part A were analysed by FACS for expression of immune markers CD4 (**b**), CD11c (**c**) and CD14 (**d**). Representative flow cytometry plots are shown in (**e**). Error bars are SEM based on nine biological replicates performed across three independent experiments. *P*-values were calculated using unpaired *t*-tests

To determine whether oestradiol deprivation and consequent increases in chemokine expression affect migration of immune cells towards tumour cells, oestradiol-deprived MCF-7 cells were co-cultured with human peripheral blood mononuclear cells (PBMCs) in a Transwell assay. Tumour cells were cultured on the bottom of each well, and PBMCs were added to the upper chamber. Conditioned media from PBMCs stimulated with PHA were used as a positive control for migration [30]. After four hours, significantly higher numbers of PBMCs were present in the bottom chamber of wells where MCF-7 cells deprived of oestradiol were cultured compared to cells grown in the presence of oestradiol (*p* = 0.002, fold change 1.3; Fig. 3a).

To assess whether the migration to the tumours was mediated by the chemokines identified in Table 2 and Fig. 2, chemokine blocking peptides were tested. Both PVNZ 112.3, which binds mainly to CXC chemokines, and PVNZ 112.6, which strongly binds CCL5 as well as other CC chemokines [31], reduced the number of PBMCs migrating towards oestrogen-deprived MCF-7 cells. Addition of the peptides led to a reduction of 36.9% and 25.8%, respectively, for PVNZ 112.3 and PVNZ 112.6 compared to oestrogen-deprived cells only (PVNZ 112.3 *p* = 0.001 and PVNZ 112.6 *p* = 0.009, Fig. 3a). This represented a mean of 23.4% and 26.4% of the one million cells added to the upper chamber migrating to the bottom chamber for PVNZ 112.3 and PVNZ 112.6, respectively, compared with 37.0% in the oestrogen-deprived vehicle control.

To assess which subsets of PBMCs migrate in response to oestrogen deprivation, we used flow cytometry to study the proportions of CD4+ T cells, CD11c+ (dendritic cells and macrophages), and CD14+ (monocytes) that had migrated to the bottom compartment of the Transwell assay described above. Significantly higher numbers of CD4+ T cells migrated towards oestrogen-deprived cells compared to cells cultured with oestradiol (*p* = 0.04, Fig. 3b). In contrast, oestrogen deprivation of MCF-7 cells significantly reduced the number of CD11c+ (*p* = 0.05) and CD14+ (*p* = 0.007) cells migrating through the transwell compared to CD11c+ or CD14+ cells migrating to MCF-7 cells receiving oestradiol (Fig. 3c–e). Collectively, these results suggest that oestrogen deprivation leads to increased production of chemokines which, in our in vitro model, appear to preferentially recruit CD4+ T cells relative to CD11c+ or CD14+ cells.

### Treatment with the NSAID aspirin reduces expression of chemokine *CCL5* and reduces migration of PBMCs towards oestrogen-deprived MCF-7 cells

**Fig. 4:**
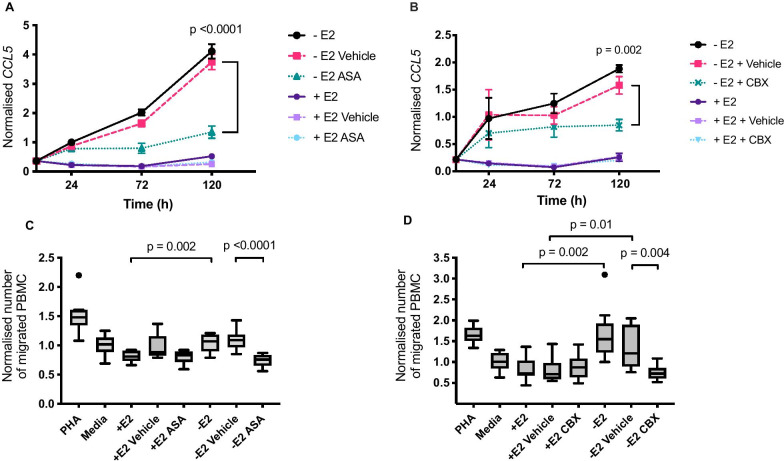
NSAIDs reduce expression of *CCL5* and reduce migration of PBMCs towards oestrogen-deprived MCF-7 cells. **a** MCF-7 cells were plated with (+E2) or without (− E2) 1 × 10^−9^ M oestradiol, treated with aspirin (1 mM) and mRNA expression of *CCL5* was determined by RT-qPCR at 0, 24, 72 and 120 h. *CCL5* expression was normalised to reference genes *FKBP15* and *PUM1.* A transwell assay was used to measure migration of PBMCs to MCF-7 cells treated with either **b** 1 mM aspirin (ASA) or **c** 10 µM celecoxib (CBX). Migration towards oestrogen supplemented (+E2) MCF-7 cells and control media (− E2) was also measured. PHA = conditioned media from PBMCs treated with phytohaemagglutinin. Data were normalised to the complete media only control (Media). Error bars are SEM based on nine biological replicates performed across three independent experiments. *P*-values were calculated using unpaired *t*-tests

CCL5 and CCL22 are strongly associated with the progression of breast cancer [35]. Our data showed that oestrogen-deprived cells produced higher levels of these chemokines and that this was associated with increased immune cell recruitment in a transwell assay. To assess whether common anti-inflammatory agents could alter chemokine expression and/or associated PBMC migration, the non-steroidal anti-inflammatory drugs (NSAIDs) aspirin and the second generation NSAID celecoxib were tested. Gene expression analysis revealed that *CCL5* expression significantly increased following oestrogen deprivation, but this effect was significantly reduced when cells were treated with aspirin (Fig. 4a, p = 0.0009 and *p* < 0.0001, 2.1 and 2.8-fold reduction at 72 and 120 h, respectively) or with celecoxib at 120 h (Fig. 4b, p = 0.002, fold reduction of 1.8). Aspirin treatment did not significantly reduce *CXCL16* and *CCL22* expression in oestrogen-deprived cells relative to vehicle, although celecoxib slightly reduced *CCL22* expression at 72 h (*p* = 0.02, 2.4-fold reduction, Additional file 6).

To assess whether the drugs affected PBMC migration in vitro, aspirin and celecoxib, a specific COX2 inhibitor, were tested in the assay in the PBMC migration assay described above. Both aspirin (1 mM) and celecoxib (10 µM) significantly reduced PBMC migration to oestradiol-deprived MCF-7 cells compared to the vehicle control (Fig. 4c and d, p < 0.0001 and *p* = 0.004, 1.5 and 1.8-fold reduction, respectively). These data indicate that immune cell migration towards oestrogen-deprived tumour cells is affected by NSAIDs and could be a result of a reduction in chemokine expression.

### Endocrine therapy increases tumour immune cell infiltrate in an orthotopic model of ER+ breast cancer in immune competent mice

**Fig. 5:**
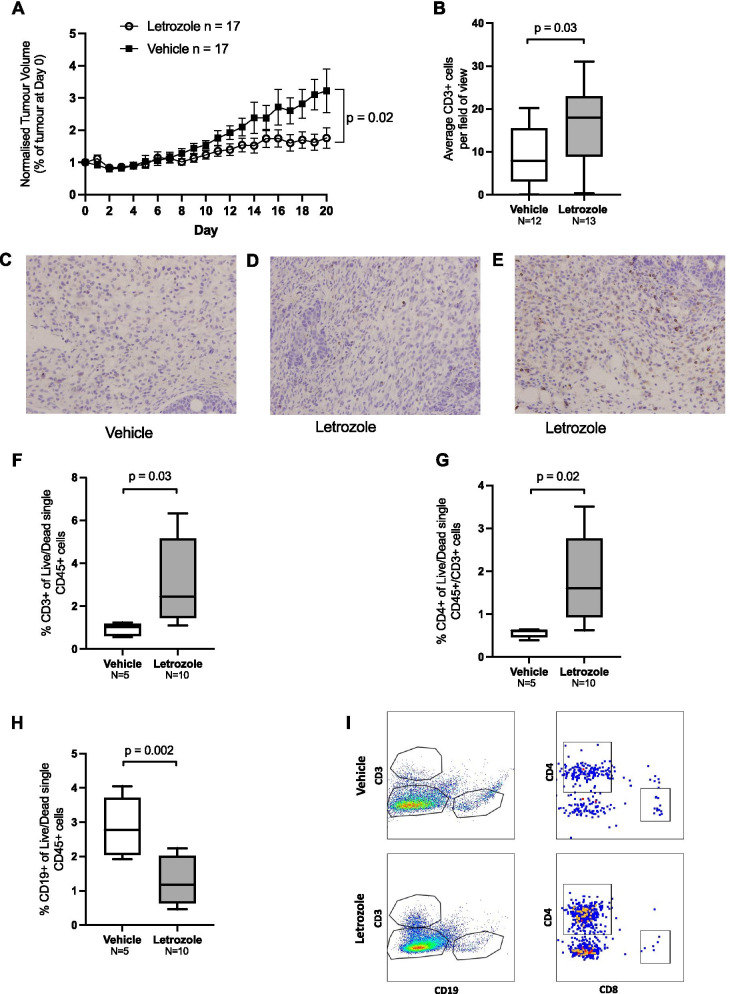
Endocrine therapy increases tumour immune cell infiltrate in an immune competent ER+ breast cancer model. **a** 129/SvEv wild-type female mice received daily treatment of 1 mg/kg letrozole for 25 days, and tumour volume was measured daily. **b** Immunohistochemistry (IHC) CD3+ staining cell counts from mammary tumours of vehicle and letrozole-treated mice. Cell counts are shown as average number of CD3+ cells in 3–7 fields at 40X magnification. **c**–**e** Representative images (40X) of low, medium and high levels CD3+ staining in stromal areas of mammary tumours. **f**–**h** FACS analysis of CD3+ (**f**), CD4+ (**g**) and CD19+ (**h**) from tumours of vehicle and letrozole-treated mice. Representative flow cytometry plots are shown in (**i**). Data are shown as percent total CD45+ lymphocytes/tumour sample. Error bars represent the mean and SEM. *P*-values were calculated using unpaired *t*-tests

Our in vitro experiments demonstrated that the number of PBMCs migrating to the bottom chamber in a migration assay could be influenced by oestrogen deprivation of MCF-7 cells. However, these in vitro assays do not include other cells present within the tumour microenvironment and are carried out over a relatively short time frame; hence, they do not fully recapitulate the tumour microenvironment. Therefore, we examined immune infiltration into tumours in a mouse model of ER+ breast cancer treated for 25 days with the AI, letrozole. In this model, immunocompetent 129/SvEv wild-type female mice were implanted with the syngeneic ER+ cell line SSM3 in the mammary fat pad [36]. After tumours reached a size of approximately 250 mm^3^, mice were ovariectomised and therapy with daily letrozole commenced to deprive the tumours of oestrogen. Letrozole treatment significantly suppressed the growth of tumours compared to the vehicle control as reflected in the tumour volume (Fig. 5a, p = 0.02).

To determine whether letrozole affected infiltration of T cells, we used immunohistochemistry to quantify the number of CD3+ T cells in the tumour after 25 days of treatment. There was a significant increase in the number of CD3+ T cells in the letrozole treatment group compared to the vehicle group (mean fold change = 1.9, *p* = 0.03) (Fig. 5b). The range across both treatment groups was an average of 0–31 CD3+ T cells per field of view. Figure 5c, d and e shows examples of low, medium and high levels of CD3 staining. This suggests that letrozole treatment may increase T cell infiltration into the tumour microenvironment.

Given the observed differences in cell populations migrating to oestrogen-deprived cells in Fig. 3, we explored additional immune populations in the tumours in response to letrozole treatment. We used flow cytometry to analyse the tumour infiltrate of broad immune cell groups of B (CD19 +) and T cell (CD3+, CD4+, CD8+) lymphocytes, and myeloid cells (CD11b+, CD11c +) in a subset of the tumours (Additional file 1) from Fig. 5a. The gating strategy for these analyses is shown in Additional file 3, and representative flow cytometry plots are shown in Fig. 5i. Consistent with the IHC data, we found a significant increase in CD3+ cells in letrozole-treated samples compared to vehicle samples (fold change = 3.34, *p* = 0.03, multiple testing corrected) (Fig. 5f). In addition, the percentage of CD4+ cells was higher (*p* = 0.02, fold change = 3.15) (Fig. 5g) and percentage CD19+ cells was lower (*p* = 0.002, fold change = 0.45) (Fig. 5h) in tumours treated with letrozole compared to vehicle-treated tumours. While there were more CD8+ T cells and CD4+ CD25+ CD127+ T-regulatory cells in letrozole-treated tumours, this was not statistically significant, and there were no significant differences between letrozole and vehicle mice in the percentages of other cells analysed (Additional file 7).

These data suggest that AI treatment of ER+ murine mammary tumours can lead to alterations in the immune composition of the tumour microenvironment. The number and percentage of CD3+ and percentage CD4+ T cells increased significantly in AI-treated samples compared to vehicle-treated samples, while a decrease in CD19+ B cells was observed.

## Discussion

In this study, we investigated the effects of oestrogen deprivation on chemokine production and immune infiltration in ER+ cancers in vitro and in vivo. Anti-oestrogen therapy is the mainstay of treatment for ER+ breast cancer, but its influence on the immune composition of breast tumours has not been fully explored. The increasing use of immunotherapies in the clinic raises the possibility that patients may receive both hormonal therapy and immunotherapy in the course of their treatment, warranting further investigation into the effects of endocrine therapy on the immune microenvironment and the potential implications for response to immunotherapy. This study provides novel data demonstrating that anti-oestrogen treatment alters immune composition in vivo and in vitro, and this can be manipulated using anti-inflammatory drugs.

Oestrogen deprivation of MCF-7 cells in vitro significantly increased production of a number of chemokines including CCL5, IL-6, IL-8 and TNF. These molecules have previously been linked to progression of breast cancer and outcome and act as chemoattractants for a diverse range of immune cells [35, 37, 38]. In addition, IL-6 has been shown to stimulate MCF-7 cells to proliferate in vitro and in vivo [39] while TNF is linked to increased invasiveness [40]. These data are consistent with our analysis of gene expression in AI-treated ER+ breast cancers from postmenopausal women where the three chemokines analysed by RT-qPCR in vitro were also upregulated in patient tumours (Table 2) and the gene expression profiles were suggestive of a broad inflammatory response to AI treatment [18]. These results suggest that oestrogen deprivation of ER+ breast cancer cells induces chemokine expression.

Oestrogen deprivation of MCF-7 cells increased migration of total PBMCs and, specifically, CD4+ T cells towards tumour cells and slightly reduced CD11c+ and CD14+ cells. Total PBMC migration was reduced by addition of either a CXC-chemokine binding protein or a CC-chemokine-binding protein indicating that migration towards tumour cells is at least partially chemokine-mediated. The effectiveness of both peptides in reducing migration suggests that a broad range of chemokines could mediate chemotaxis. However, it should be noted that both peptides have some binding to CCL5 [31]. *CCL5* transcript levels were increased in human tumours treated with AIs and protein levels of CCL5 increased 35-fold 120 h after oestrogen deprivation in cultured cells, placing CCL5 as a likely key mediator of the migration response. Elevated CCL5 levels are correlated with progression of breast cancer [35] as well as increasing the metastatic potential of MDA-MB-231 cells in vivo [41]. CCL5 is a ligand for C–C chemokine receptors CCR1, 3, 4 and 5 in both mice and humans and hence has the potential to recruit both monocytes and macrophages [24]. In addition, high *CCL5* expression has been associated with recruitment of CD8+ T cells, CD4+ activated T cells, NK activated cells and macrophages M1 [42, 43].

The immune alterations observed in vitro were broadly recapitulated in our murine model of ER+ breast cancer where the total number of CD3+ cells and the proportion of CD4+ T cells was higher in letrozole-treated tumours when compared to vehicle-treated tumours (Fig. 5). A related observation has recently been made in ER+ patient tumours where an increase in tumour-infiltrating lymphocytes (TILs) was observed after treatment with letrozole [17]. We also observed a non-significant trend for higher levels of CD4+ CD25+ CD127- (Tregs) and CD8+ cells in letrozole-treated SSM3 tumours (Additional File 7). High levels of Tregs in ER+ have previously been shown to confer reduced overall survival and relapse-free survival suggesting that these cells could have a negative impact on outcome [44]. Taken together, these results support the concept that oestrogen deprivation of tumours induces changes in infiltrating immune cells in the tumour microenvironment. However, the specific consequences of these changes in the context of endocrine-therapy treated breast cancer are not well understood. While numerous studies have demonstrated that increased TILs are associated with improved outcomes in patients with triple-negative and Her2-positive breast cancer, no significant prognostic value has been found for TILs in ER+ Her2- disease [45], particularly in patients who do not receive chemotherapy [46]. Similarly, CD8+ T-cells have been associated with a significantly reduced relative risk of death from breast cancer in ER- and in HER2-enriched tumours, but not in ER+ cases [47, 48]. In addition, higher numbers of naive CD4+ T cells have been associated with poor prognosis in human breast cancer and these CD4+ cells are likely the main source of immunosuppressive Tregs in breast cancer [49]. The lack of clarity surrounding this issue is the ER-cohorts used in these studies which have been largely derived from patients treated with adjuvant chemotherapy. Given the well-established direct effects of chemotherapy on the immune system, it seems likely that the immune modification and long-term outcome will differ in patients treated with endocrine therapy alone when compared to patients who receive additional adjuvant treatments, particularly chemotherapy.

There are a number of potential mechanisms through which oestrogen deprivation could lead to the chemokine production which appears to drive immune cell infiltration in this study. Oestrogen deprivation and aromatase inhibitor treatment are associated with alterations in the expression of thousands of genes [18, 20, 50] which may reflect the many potential cellular pathways that mediate the effect of these treatments on chemokine expression. More specifically, oestradiol has also been shown to repress mRNA expression of chemokines in other tissues including *CCL5* in a mouse model of autoimmune encephalomyelitis [51] and a range of inflammatory cytokines in Myasthenia gravis [52]. The mechanism driving the effect in these systems is poorly understood, but type 1 interferon production appears to be controlled by oestrogen in some cell types [52]. Additional drivers of the pro-inflammatory phenotype in response to oestrogen deprivation could come through induction of apoptosis, a well-established precipitant of inflammation [53]. Also, the effectiveness of the specific cyclooxygenase 2 (COX2) inhibitor celecoxib—which directly inhibits COX2 which produces inflammatory prostaglandins [54]—in suppressing chemokine production suggests that its main product prostaglandin E2 may play a role in driving or potentiating this process. High COX2 activity and PGE2 levels are associated with TNF and IL-6 [10] which could generate a proinflammatory environment driving the production of a range of chemokines. COX-2 is expressed in approximately 70% of breast cancers [55] and can be elevated by cytokines, growth factors and is involved in inflammatory processes. Prostaglandins in turn have been demonstrated to elevate cAMP causing upregulation of aromatase and increased local production of oestrogen [56]. Stromal and immune cells also express ER and aromatase [16].

Oestrogen deprivation may also have a more direct effect on immune cells. ERα is expressed in most cells of the immune system, including hematopoietic cells, bone marrow, murine splenic DC and peritoneal macrophages [15]; hence, it is possible that some of the immune effects seen in human patients and our murine model are the result of direct regulation of immune cells in response to oestrogen withdrawal. Supporting a direct effect are recent findings examining the effect of selective ER downregulators (SERDs) in an ER-4T1 murine model [57]. This study showed increased numbers of CD8+ cells by IHC and decreased CD4+ CD25+ FoxP3+ Tregs in ER-tumours in mice treated with SERDs. While our study showed small non-significant increases in CD8+ T cells and CD4+ CD25+ CD127+ T-regulatory cells in ER+ tumours, it is possible that the tumour-independent effects seen in the ER-model may interact with the tumour chemokine-mediated effects via chemokine recruitment that we have demonstrated in vitro. What implications the effects on different cell types could have for other types of endocrine therapy is also unknown. A previous study involving treatment of murine tumours with tamoxifen suggested tamoxifen abrogated macrophage influx by reducing CCL2/CCL5 levels and reversed macrophage activation [58]. However, differences in the ER expression levels in the models used make it difficult to assess how generally applicable these findings are.

Irrespective of the mechanism through which immune cell recruitment is mediated, this work has implications for treatment selection for patients receiving endocrine therapy. Our previous work has suggested that patients with high levels of immune involvement have poorer responses when treated with aromatase inhibitor monotherapy compared to patients with lower immune involvement [18, 19]. Large analyses have also revealed that immune infiltration does not predict better outcome in the ER+ Her2-subgroup [45, 47, 48]. While there is little doubt that endocrine therapy confers a net benefit for the ER+ patient group, the most appropriate way to manage the immune infiltration generated by endocrine monotherapy remains to be determined. In retrospective analyses, aspirin intake has been associated with a reduction in risk of disease recurrence and breast cancer-related death [59–62] suggesting that anti-inflammatory therapy may have some benefits in breast cancer patients. However, in patients receiving additional therapy such as immunotherapy, it is possible that the additional immune infiltration could improve outcomes if the immunosuppressive, inflammatory response could be re-directed to an anti-tumour one. Further pre-clinical studies and clinical trials need to be carried out to assess which is most appropriate for specific patient groups.

## Conclusions

Our findings demonstrate that withdrawal of oestrogen increases chemokine production by ER+ breast cancer cells. The chemokines produced drive migration of immune cells towards tumour cells in vitro, and tumour infiltration after withdrawal of oestrogen is also seen in our murine model of ER+ breast cancer. These observations are supported by our analysis of gene expression data from aromatase inhibitor-treated ER+ breast cancer patients and previously published work demonstrating that TILs increase following aromatase inhibitor treatment [17]. Further characterisation of the immune response to endocrine therapy in patients is required to inform the timing and combination of treatments to achieve the best outcome for patients.

## Supplementary Information

**Additional file 1**. Venn diagram showing overlap of sample groups used for SSM3 Mouse tumour analysis. Insufficient tumour material was available for both flow analysis and IHC, requiring separate samples.

**Additional file 2**. Flow Cytometry Gating Strategy for human PBMC Analysis. Plots show (A) Live/Dead, (B) Single cells, (C) Lymphocytes, (D) CD45+, (E) CD3+, (F) CD11+, (G) CD14+, (H) CD19+ and (I) CD4+ and CD8+ gates.

**Additional file 3**. Flow Cytometry Gating Strategy for Mouse Tumour Analysis. Plots show (A) CD45+ Live/Dead, (B) Single cells, (C) Lymphocytes and innate cells, (D) CD25+/CD127-, (E) CD4+ and CD8+, (F) CD3+ and CD19+, (G) CD11+ and CD11c+ gates.

**Additional file 4**. Chemokine expression of oestrogen receptor negative cell line SKBR3 is not altered by oestrogen deprivation. SKBR3 cells were cultured with (+ E2) or without (-E2) 1 × 10^−9^ M oestradiol and mRNA expression of three chemokines (A) *CCL5*, (B) *CXCL16* and (C) *CCL22* was determined by RT-qPCR at 0, 24, 72 and 120 h. Data are normalised to three reference genes, *FKBP15, TBP* and *PUM1*. Error bars are SEM based on three biological replicates performed across one experiment.

**Additional file 5**. Cytokine secretion in SKBR3 human breast cancer cells is not altered by oestrogen deprivation. (A) CCL5, (B) TNF, (C) IL-18, (D) CCL22 and (E) IL-6. SKBR3 cells were with cultured with (+ E2) or without (− E2) 1 × 10^−9^ M oestradiol and cell supernatant was collected at 0, 24, 72 and 120 h and analysed with a multiplex assay. Data are normalised to the number of cells in each well. Error bars are SEM based on six biological replicates performed across three independent experiments. *P*-values were calculated using unpaired *t*-tests.

**Additional file 6**. Aspirin does not alter expression of (A) *CXCL16* or (B) *CCL22* in MCF-7 cells without oestrogen. MCF-7 cells were plated with (+ E2) or without (− E2) 1 × 10^−9^ M oestradiol and treated with aspirin (1 mM). mRNA expression of *CCL22* and *CXCL16* was determined by RT-qPCR at 0, 24, 72 and 120 h and normalised to reference genes *FKBP15* and *PUM1*. Error bars are SEM based on nine biological replicates performed across three independent experiments. *P*-values were calculated using unpaired *t*-tests.

**Additional file 7**. Analysis of remaining immune cell types in SSM3 Mouse tumours as determined by Flow Cytometry. (A) % CD45+ cells, (B) % CD8+, (C) % of CD11b+, (D) % of CD11c+, (E) % of CD11b-/CD11c+ and (F) % of CD4+ CD25+ CD127- cells. Error bars are the mean and SEM, and all cell types were non-significant as determined by unpaired *t*-test.

## Data Availability

The datasets generated during and/or analysed during the current study are available from the corresponding author on reasonable request.

## Abbreviations

AIs: Aromatase inhibitors
CCL5: C–C chemokine ligand 5
CCL22: C–C chemokine ligand 22
COX2: Cyclooxygenase 2
CXCL16: C-X-C Motif Chemokine Ligand 16
DCC: Dextran charcoal stripped bovine serum
ER+: Oestrogen receptor positive
FACS: Flow cytometry
IHC: Immunohistochemistry
NBF: Neutral buffered formalin
NSAID: Non-steroidal anti-inflammatory drugs
PBMCs: Peripheral blood mononuclear cells
PHA: Phytohemagglutinin
PVNZ: Parapoxvirus chemokine binding proteins
RT-qPCR: Reverse transcriptase quantitative PCR
SERDs: Selective ER downregulators
STR: Short Tandem Repeat
TILs: Tumour-infiltrating lymphocytes

## References

[1] Bray F, Ferlay J, Soerjomataram I, Siegel RL, Torre LA, Jemal A (2018). Global cancer statistics 2018: GLOBOCAN estimates of incidence and mortality worldwide for 36 cancers in 185 countries. CA A Cancer J Clin.

[2] Dodson A, Parry S, Ibrahim M, Bartlett JM, Pinder S, Dowsett M (2018). Breast cancer biomarkers in clinical testing: analysis of a UK national external quality assessment scheme for immunocytochemistry and in situ hybridisation database containing results from 199 300 patients. J Pathol Clin Res.

[3] DeSantis CE, Ma J, Gaudet MM, Newman LA, Miller KD, Goding Sauer A (2019). Breast cancer statistics, 2019. CA Cancer J Clin.

[4] Fabian CJ (2007). The what, why and how of aromatase inhibitors: hormonal agents for treatment and prevention of breast cancer. Int J Clin Pract.

[5] Darbre PD, Curtis S, King RJ (1984). Effects of estradiol and tamoxifen on human breast cancer cells in serum-free culture. Cancer Res.

[6] Smith IE, Dowsett M, Ebbs SR, Dixon JM, Skene A, Blohmer J-U (2005). Neoadjuvant treatment of postmenopausal breast cancer with anastrozole, tamoxifen, or both in combination: the immediate preoperative anastrozole, tamoxifen, or combined with tamoxifen (IMPACT) multicenter double-blind randomized trial. J Clin Oncol.

[7] Mouridsen H, Gershanovich M, Sun Y, Pérez-Carrión R, Boni C, Monnier A (2001). Superior efficacy of letrozole versus tamoxifen as first-line therapy for postmenopausal women with advanced breast cancer: results of a phase III study of the international letrozole breast cancer group. J Clin Oncol.

[8] Robertson JFR, Bondarenko IM, Trishkina E, Dvorkin M, Panasci L, Manikhas A (2016). Fulvestrant 500 mg versus anastrozole 1 mg for hormone receptor-positive advanced breast cancer (FALCON): an international, randomised, double-blind, phase 3 trial. The Lancet.

[9] Nabholtz JM, Buzdar A, Pollak M, Harwin W, Burton G, Mangalik A (2000). Anastrozole Is superior to tamoxifen as first-line therapy for advanced breast cancer in postmenopausal women: results of a North American multicenter randomized trial. J Clin Oncol.

[10] Colleoni M, Sun Z, Price KN, Karlsson P, Forbes JF, Thürlimann B (2016). Annual hazard rates of recurrence for breast cancer during 24 years of follow-up: results from the international breast cancer study group trials I to V. J Clin Oncol.

[11] Pan H, Gray R, Braybrooke J, Davies C, Taylor C, McGale P (2017). 20-year risks of breast-cancer recurrence after stopping endocrine therapy at 5 years. N Engl J Med.

[12] Jacobson DL, Gange SJ, Rose NR, Graham NMH (1997). Epidemiology and estimated population burden of selected autoimmune diseases in the United States. Clin Immunol Immunopathol.

[13] Abdullah M, Chai P-S, Chong M-Y, Tohit ERM, Ramasamy R, Pei CP (2012). Gender effect on in vitro lymphocyte subset levels of healthy individuals. Cell Immunol.

[14] Engler RJM (2008). Half- vs full-dose trivalent inactivated influenza vaccine (2004–2005). Arch Intern Med.

[15] Khan D, Ansar Ahmed S. The immune system is a natural target for estrogen action: opposing effects of estrogen in two prototypical autoimmune diseases. Front Immunol. 2016;6. 10.3389/fimmu.2015.00635 PMC4701921 26779182

[16] Rothenberger N, Somasundaram A, Stabile L (2018). The role of the Estrogen pathway in the tumor microenvironment. Int J Mol Sci.

[17] Skriver SK, Jensen M-B, Knoop AS, Ejlertsen B, Laenkholm A-V (2020). Tumour-infiltrating lymphocytes and response to neoadjuvant letrozole in patients with early oestrogen receptor-positive breast cancer: analysis from a nationwide phase II DBCG trial. Breast Cancer Res.

[18] Dunbier AK, Ghazoui Z, Anderson H, Salter J, Nerurkar A, Osin P (2013). Molecular profiling of aromatase inhibitor-treated postmenopausal breast tumors identifies immune-related correlates of resistance. Clin Cancer Res.

[19] Gao Q, Patani N, Dunbier AK, Ghazoui Z, Zvelebil M, Martin L-A (2014). Effect of aromatase inhibition on functional gene modules in Estrogen receptor-positive breast cancer and their relationship with antiproliferative response. Clin Cancer Res.

[20] Gao Q, López-Knowles E, Cheang MCU, Morden J, Ribas R, Sidhu K (2019). Impact of aromatase inhibitor treatment on global gene expression and its association with antiproliferative response in ER+ breast cancer in postmenopausal patients. Breast Cancer Res.

[21] Jin T, Xu X, Hereld D (2008). Chemotaxis, chemokine receptors and human disease. Cytokine.

[22] Sokol CL, Luster AD (2015). The Chemokine System in Innate Immunity. Cold Spring Harbor Perspect Biol.

[23] Allavena P, Germano G, Marchesi F, Mantovani A (2011). Chemokines in cancer related inflammation. Exp Cell Res.

[24] Nagarsheth N, Wicha MS, Zou W (2017). Chemokines in the cancer microenvironment and their relevance in cancer immunotherapy. Nat Rev Immunol.

[25] Smith IE, Walsh G, Skene A, Llombart A, Mayordomo JI, Detre S (2007). A phase II placebo-controlled trial of neoadjuvant anastrozole alone or with gefitinib in early breast cancer. J Clin Oncol.

[26] Du P, Kibbe WA, Lin SM (2008). Lumi: a pipeline for processing Illumina microarray. Bioinformatics.

[27] Molecular profiling of aromatase inhibitor-treated post-menopausal breast tumors identifies immunerelated correlates of resistance [Internet]. 2020. Available from: https://www.ncbi.nlm.nih.gov/geo/query/acc.cgi?acc=GSE153470.

[28] Team DRSB-AD. BRB-ArrayTools [Available from: https://brb.nci.nih.gov/BRB-ArrayTools/.

[29] Chang JT, Nevins JR (2006). GATHER: a systems approach to interpreting genomic signatures. Bioinformatics.

[30] Carr MW, Roth SJ, Luther E, Rose SS, Springer TA (1994). Monocyte chemoattractant protein 1 acts as a T-lymphocyte chemoattractant. Proc Natl Acad Sci.

[31] Sharif S, Ueda N, Nakatani Y, Wise LM, Clifton S, Lateef Z (2019). Chemokine-binding proteins encoded by Parapoxvirus of red deer of New Zealand display evidence of gene duplication and divergence of ligand specificity. Front Microbiol.

[32] Schlecker E, Stojanovic A, Eisen C, Quack C, Falk CS, Umansky V (2012). Tumor-infiltrating monocytic myeloid-derived suppressor cells mediate CCR5-dependent recruitment of regulatory T cells favoring tumor growth. J Immunol.

[33] Gobert M, Treilleux I, Bendriss-Vermare N, Bachelot T, Goddard-Leon S, Arfi V (2009). Regulatory T cells recruited through CCL22/CCR4 are selectively activated in lymphoid infiltrates surrounding primary breast tumors and lead to an adverse clinical outcome. Can Res.

[34] Curiel TJ, Coukos G, Zou L, Alvarez X, Cheng P, Mottram P (2004). Specific recruitment of regulatory T cells in ovarian carcinoma fosters immune privilege and predicts reduced survival. Nat Med.

[35] Luboshits G, Shina S, Kaplan O, Engelberg S, Nass D, Lifshitz-Mercer B (1999). Elevated expression of the CC chemokine regulated on activation, normal T cell expressed and secreted (RANTES) in advanced breast carcinoma. Can Res.

[36] Chan SR, Vermi W, Luo J, Lucini L, Rickert C, Fowler AM (2012). STAT1-deficient mice spontaneously develop estrogen receptor α-positive luminal mammary carcinomas. Breast Cancer Res.

[37] Li YQ, Liu FF, Zhang XM, Guo XJ, Ren MJ, Fu L (2013). Tumor secretion of CCL22 activates intratumoral Treg infiltration and is independent prognostic predictor of breast cancer. PLOS ONE.

[38] Ma Y, Ren Y, Dai ZJ, Wu CJ, Ji YH, Xu J (2017). IL-6, IL-8 and TNF-alpha levels correlate with disease stage in breast cancer patients. Adv Clin Exp Med.

[39] Sasser AK, Sullivan NJ, Studebaker AW, Hendey LF, Axel AE, Hall BM (2007). Interleukin-6 is a potent growth factor for ER-α-positive human breast cancer. FASEB J.

[40] Yin Y, Chen X, Shu Y (2009). Gene expression of the invasive phenotype of TNF-alpha-treated MCF-7 cells. Biomed Pharmacother.

[41] Karnoub AE, Dash AB, Vo AP, Sullivan A, Brooks MW, Bell GW (2007). Mesenchymal stem cells within tumour stroma promote breast cancer metastasis. Nature.

[42] Araujo JM, Gomez AC, Aguilar A, Salgado R, Balko JM, Bravo L (2018). Effect of CCL5 expression in the recruitment of immune cells in triple negative breast cancer. Sci Rep.

[43] Liu J, Guan X, Ma X (2005). Interferon regulatory factor 1 is an essential and direct transcriptional activator for interferon {gamma}-induced RANTES/CCl5 expression in macrophages. J Biol Chem.

[44] Bates GJ, Fox SB, Han C, Leek RD, Garcia JF, Harris AL (2006). Quantification of regulatory T cells enables the identification of high-risk breast cancer patients and those at risk of late relapse. J Clin Oncol.

[45] Savas P, Salgado R, Denkert C, Sotiriou C, Darcy PK, Smyth MJ (2016). Clinical relevance of host immunity in breast cancer: from TILs to the clinic. Nat Rev Clin Oncol.

[46] Criscitiello C, Vingiani A, Maisonneuve P, Viale G, Viale G, Curigliano G (2020). Tumor-infiltrating lymphocytes (TILs) in ER+/HER2− breast cancer. Breast Cancer Res Treat.

[47] Burugu S, Asleh-Aburaya K, Nielsen TO (2017). Immune infiltrates in the breast cancer microenvironment: detection, characterization and clinical implication. Breast Cancer.

[48] Byrne A, Savas P, Sant S, Li R, Virassamy B, Luen SJ (2020). Tissue-resident memory T cells in breast cancer control and immunotherapy responses. Nat Rev Clin Oncol.

[49] Su S, Liao J, Liu J, Huang D, He C, Chen F (2017). Blocking the recruitment of naive CD4(+) T cells reverses immunosuppression in breast cancer. Cell Res.

[50] Yamaga R, Ikeda K, Horie-Inoue K, Ouchi Y, Suzuki Y, Inoue S (2013). RNA sequencing of MCF-7 breast cancer cells Identifies Novel Estrogen-responsive genes with functional estrogen receptor-binding sites in the vicinity of their transcription start sites. Hormones Cancer.

[51] Matejuk A, Adlard K, Zamora A, Silverman M, Vandenbark AA, Offner H (2001). 17 beta-estradiol inhibits cytokine, chemokine, and chemokine receptor mRNA expression in the central nervous system of female mice with experimental autoimmune encephalomyelitis. J Neurosci Res.

[52] Dragin N, Nancy P, Villegas J, Roussin R, Le Panse R, Berrih-Aknin S (2017). Balance between Estrogens and proinflammatory cytokines regulates chemokine production involved in thymic germinal center formation. Sci Rep.

[53] Musgrove EA, Sutherland RL (2009). Biological determinants of endocrine resistance in breast cancer. Nat Rev Cancer.

[54] Herschman HR (1996). Prostaglandin synthase 2. Biochim Biophys Acta.

[55] Shim JY (2003). Overexpression of cyclooxygenase-2 is associated with breast carcinoma and its poor prognostic factors. Mod Pathol.

[56] Zhao Y, Agarwal VR, Mendelson CR, Simpson ER (1996). Estrogen biosynthesis proximal to a breast tumor is stimulated by PGE2 via cyclic AMP, leading to activation of promoter II of the CYP19 aromatase gene. Endocrinology.

[57] Márquez-Garbán DC, Deng G, Comin-Anduix B, Garcia AJ, Xing Y, Chen HW (2019). Antiestrogens in combination with immune checkpoint inhibitors in breast cancer immunotherapy. J Steroid Biochem Mol Biol.

[58] Svensson S, Abrahamsson A, Rodriguez GV, Olsson A-K, Jensen L, Cao Y (2015). CCL2 and CCL5 are novel therapeutic targets for Estrogen-dependent breast cancer. Clin Cancer Res.

[59] Fraser DM, Sullivan FM, Thompson AM, McCowan C (2014). Aspirin use and survival after the diagnosis of breast cancer: a population-based cohort study. Br J Cancer.

[60] Blair CK, Sweeney C, Anderson KE, Folsom AR (2007). NSAID use and survival after breast cancer diagnosis in post-menopausal women. Breast Cancer Res Treat.

[61] Holmes MD, Chen WY, Li L, Hertzmark E, Spiegelman D, Hankinson SE (2010). Aspirin intake and survival after breast cancer. J Clin Oncol.

[62] Liu J, Zheng F, Yang M, Wu X, Liu A (2021). Effect of aspirin use on survival benefits of breast cancer patients: A meta-analysis. Med Baltim.

