# Supplementary figures and images for: Oestrogen deprivation induces chemokine production and immune cell recruitment in in vitro and in vivo models of oestrogen receptor-positive breast cancer
Source: Breast Cancer Res. 2021 Oct 3;23:95. doi: 10.1186/s13058-021-01472-1 (PMC8489094; doi:10.1186/s13058-021-01472-1)

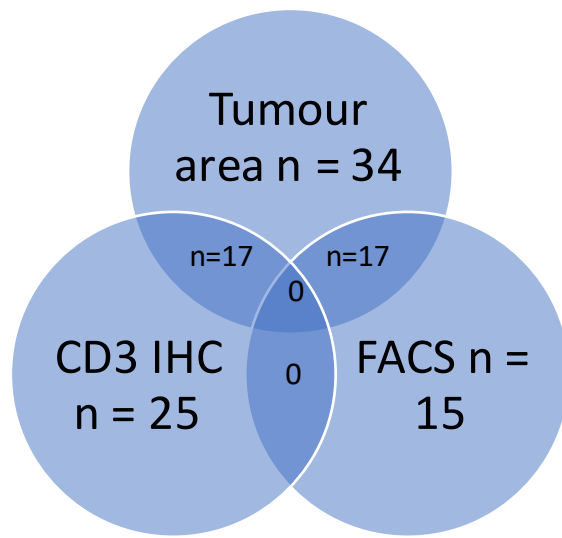

Supplement: Supplementary file 1 — Additional file 1. Venn diagram showing overlap of sample groups used for SSM3 Mouse tumour analysis. Insufficient tumour material was available for both flow analysis and IHC, requiring separate samples. [file 13058_2021_1472_MOESM1_ESM.pdf]

FACS Gating strategy for PBMCs.

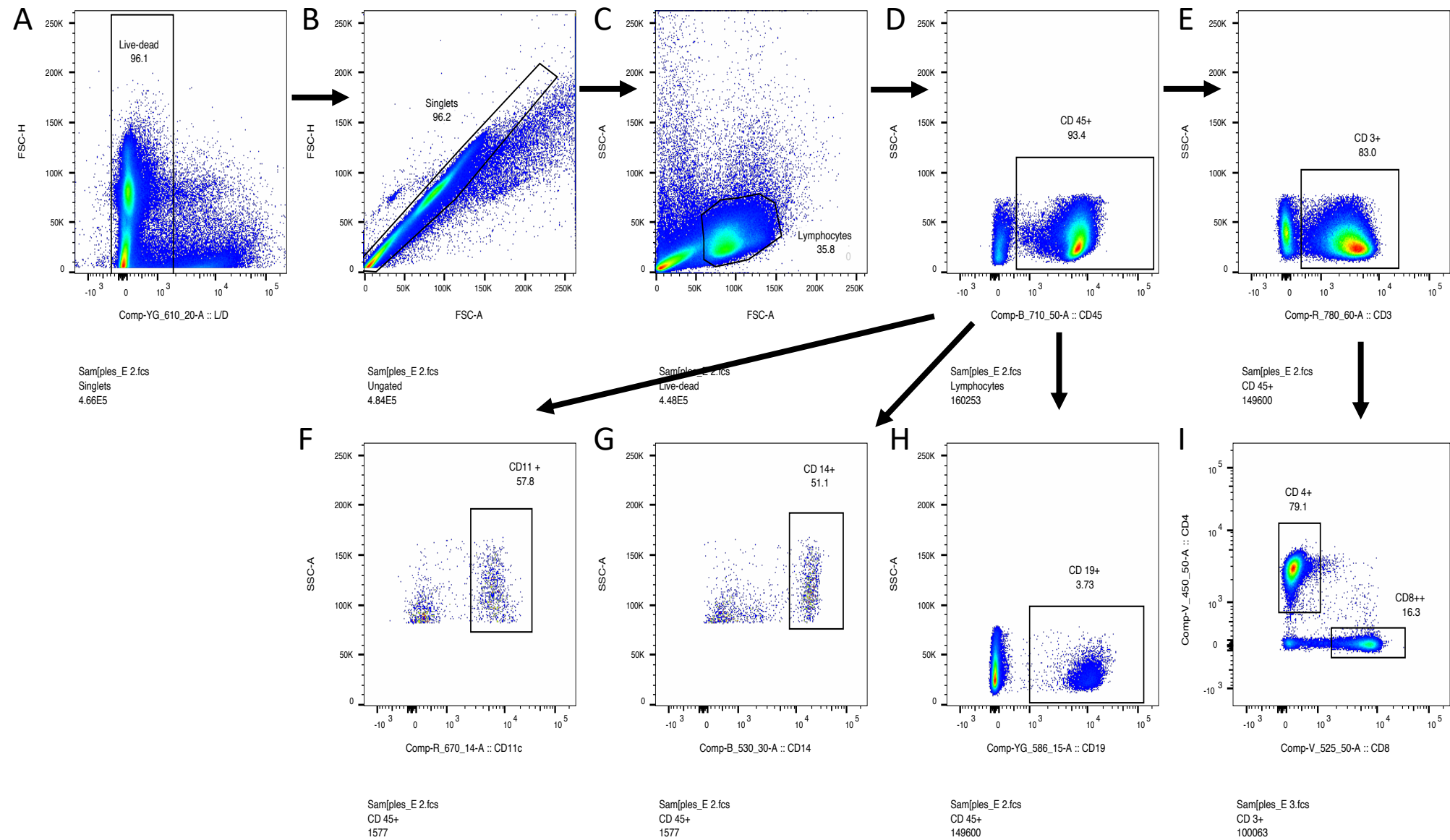

Supplement: Supplementary file 2 — Additional file 2. Flow Cytometry Gating Strategy for human PBMC Analysis. Plots show (A) Live/Dead, (B) Single cells, (C) Lymphocytes, (D) CD45+, (E) CD3+, (F) CD11+, (G) CD14+, (H) CD19+ and (I) CD4+ and CD8+ gates. [file 13058_2021_1472_MOESM2_ESM.pdf]

# FACS Gating Strategy

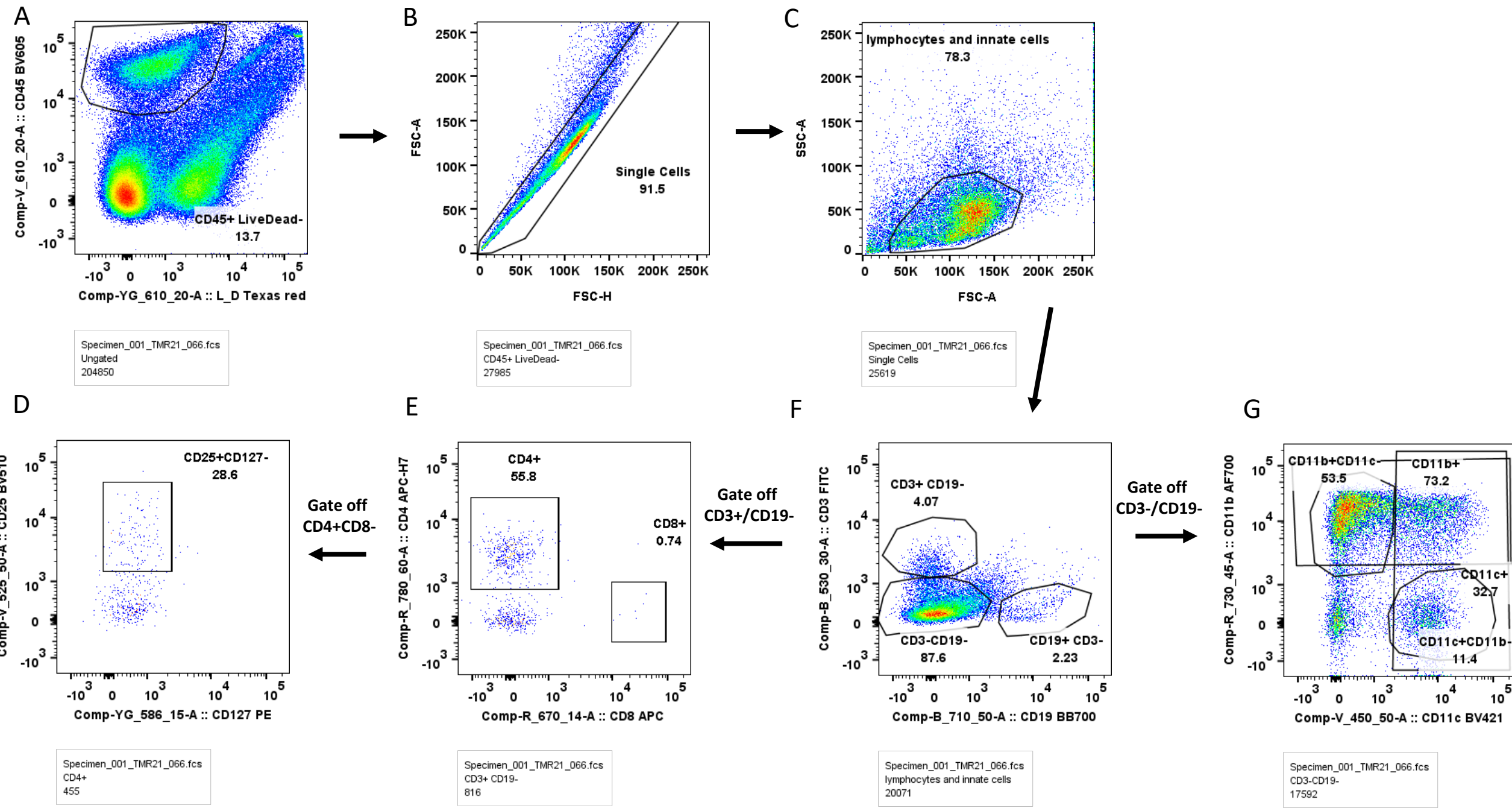

Supplement: Supplementary file 3 — Additional file 3. Flow Cytometry Gating Strategy for Mouse Tumour Analysis. Plots show (A) CD45+ Live/Dead, (B) Single cells, (C) Lymphocytes and innate cells, (D) CD25+/CD127-, (E) CD4+ and CD8+, (F) CD3+ and CD19+, (G) CD11+ and CD11c+ gates. [file 13058_2021_1472_MOESM3_ESM.pdf]

**A**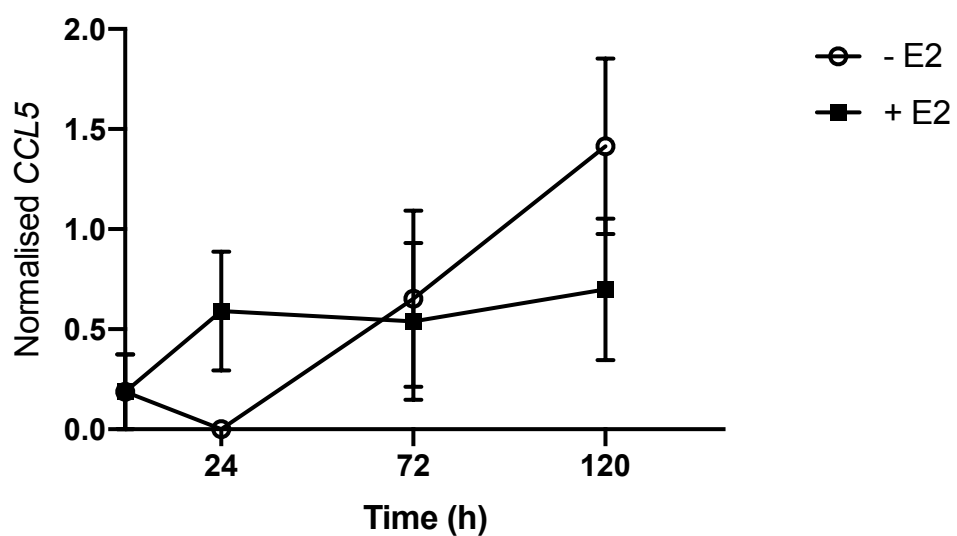**B**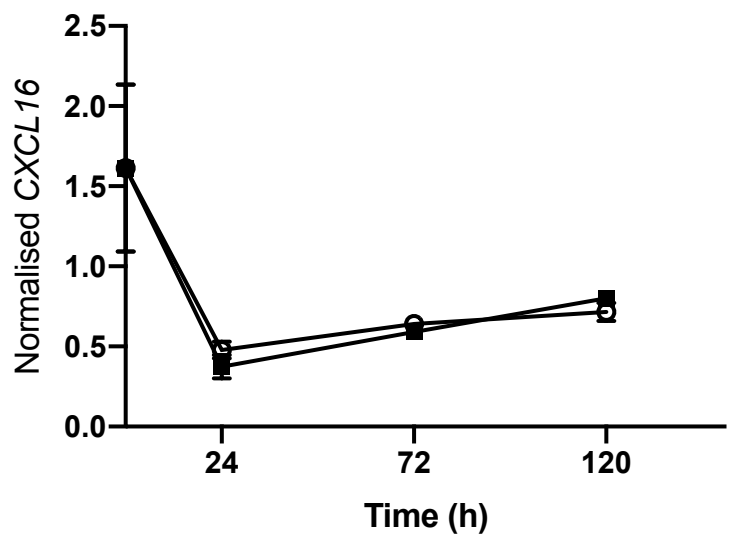**C**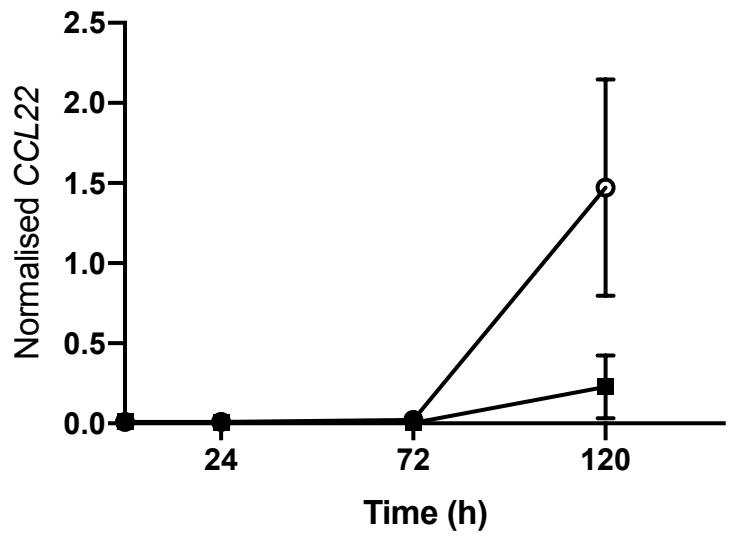

Supplement: Supplementary file 4 — Additional file 4. Chemokine expression of oestrogen receptor negative cell line SKBR3 is not altered by oestrogen deprivation. SKBR3 cells were cultured with (+ E2) or without (-E2) 1 × 10^−9^ M oestradiol and mRNA expression of three chemokines (A) CCL5, (B) CXCL16 and (C) CCL22 was determined by RT-qPCR at 0, 24, 72 and 120 h. Data are normalised to three reference genes, FKBP15, TBP and PUM1. Error bars are SEM based on three biological replicates performed across one experiment. [file 13058_2021_1472_MOESM4_ESM.pdf]

**A**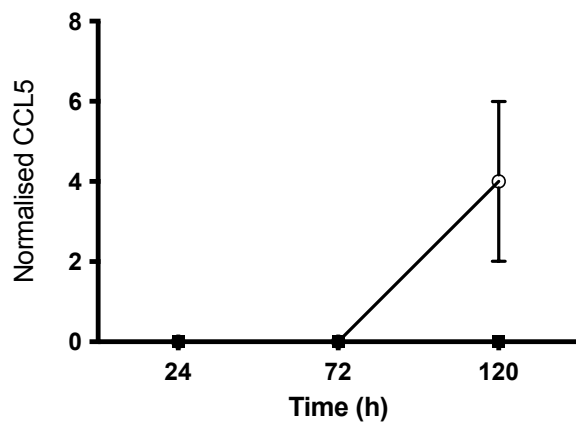**B**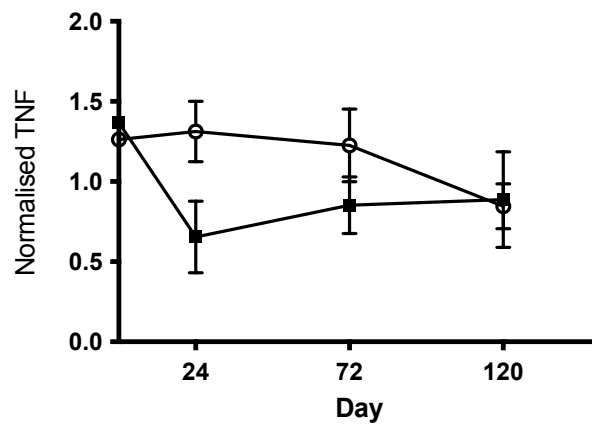**C**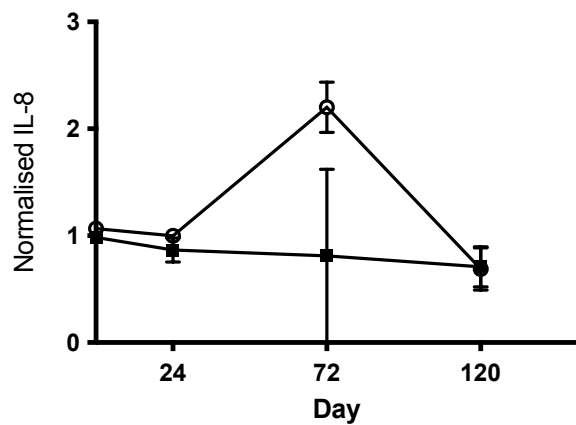**D**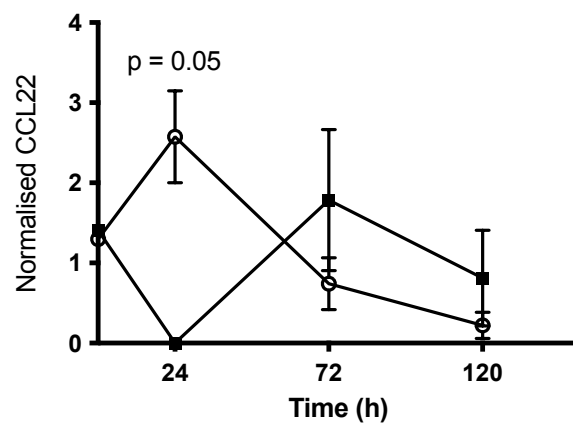**E**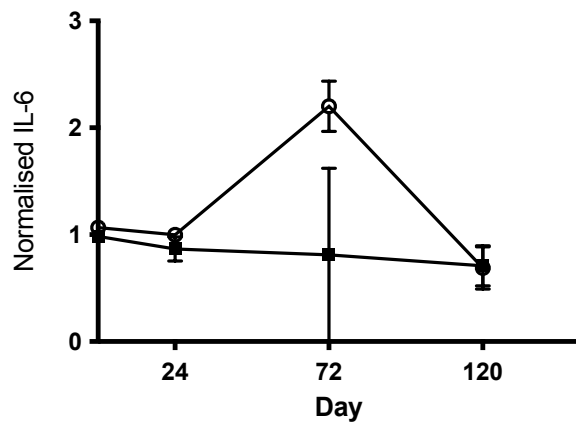

○ SKBR3-E2  
■ SK E2

Supplement: Supplementary file 5 — Additional file 5. Cytokine secretion in SKBR3 human breast cancer cells is not altered by oestrogen deprivation. (A) CCL5, (B) TNF, (C) IL-18, (D) CCL22 and (E) IL-6. SKBR3 cells were with cultured with (+ E2) or without (− E2) 1 × 10^−9^ M oestradiol and cell supernatant was collected at 0, 24, 72 and 120 h and analysed with a multiplex assay. Data are normalised to the number of cells in each well. Error bars are SEM based on six biological replicates performed across three independent experiments. P-values were calculated using unpaired t-tests. [file 13058_2021_1472_MOESM5_ESM.pdf]

A

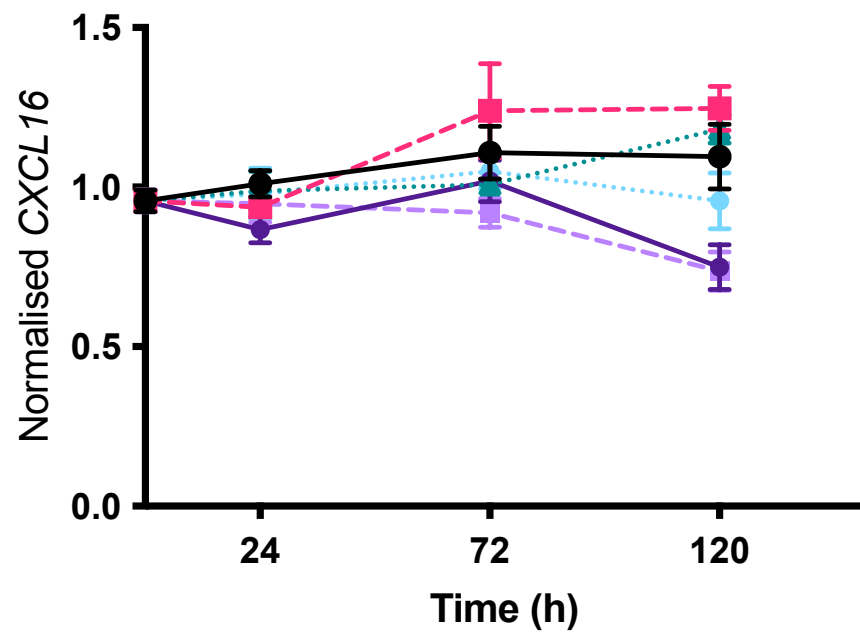

B

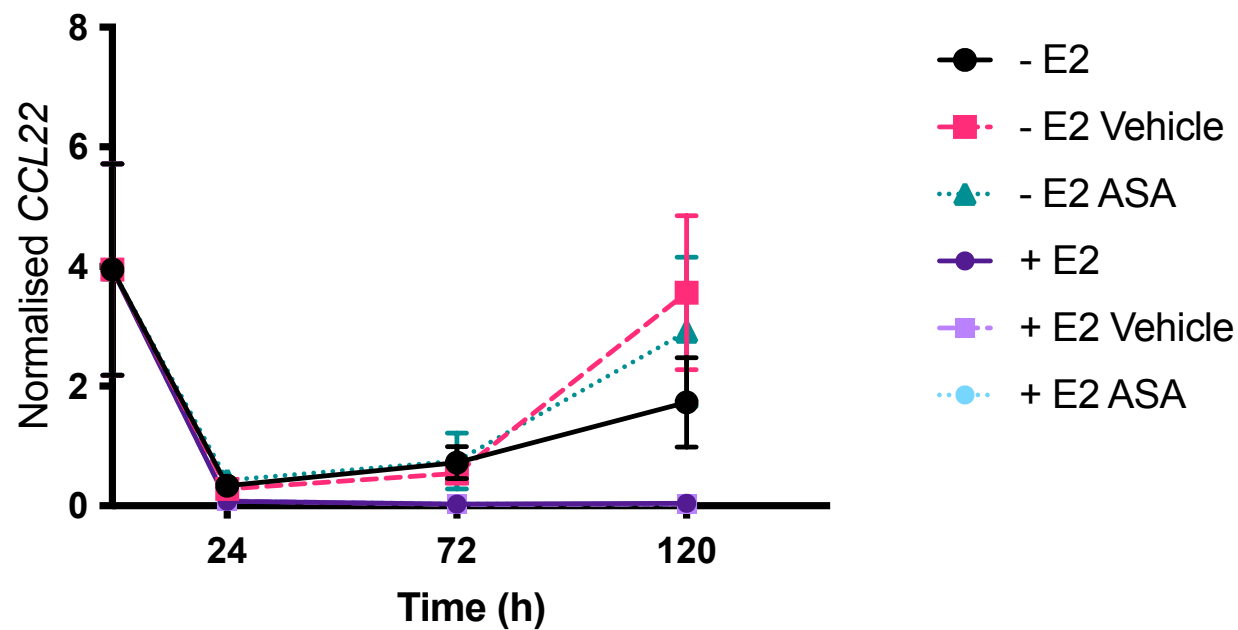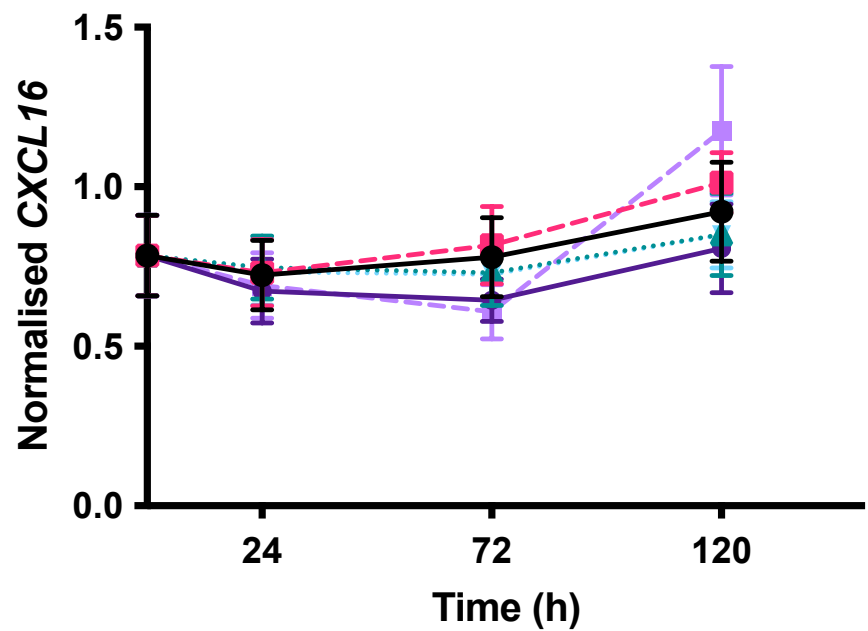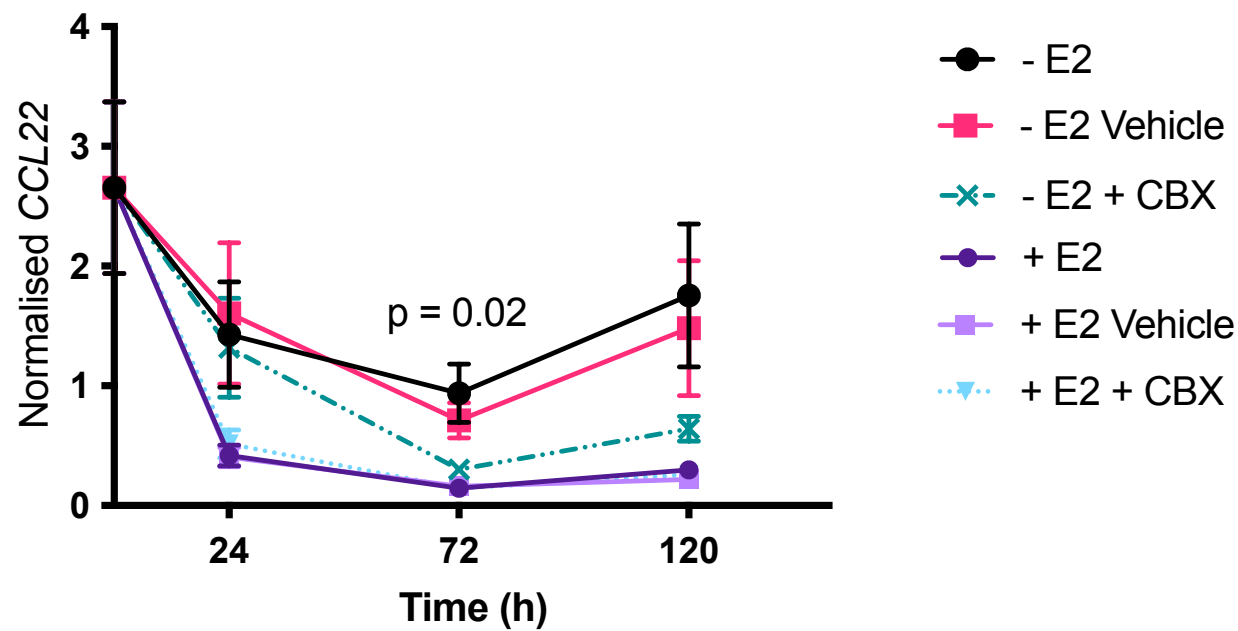

Supplement: Supplementary file 6 — Additional file 6. Aspirin does not alter expression of (A) CXCL16 or (B) CCL22 in MCF-7 cells without oestrogen. MCF-7 cells were plated with (+ E2) or without (− E2) 1 × 10^−9^ M oestradiol and treated with aspirin (1 mM). mRNA expression of CCL22 and CXCL16 was determined by RT-qPCR at 0, 24, 72 and 120 h and normalised to reference genes FKBP15 and PUM1. Error bars are SEM based on nine biological replicates performed across three independent experiments. P-values were calculated using unpaired t-tests. [file 13058_2021_1472_MOESM6_ESM.pdf]

**A**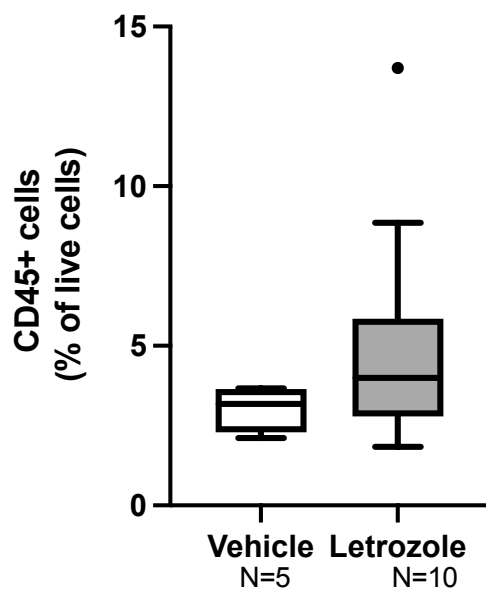**B**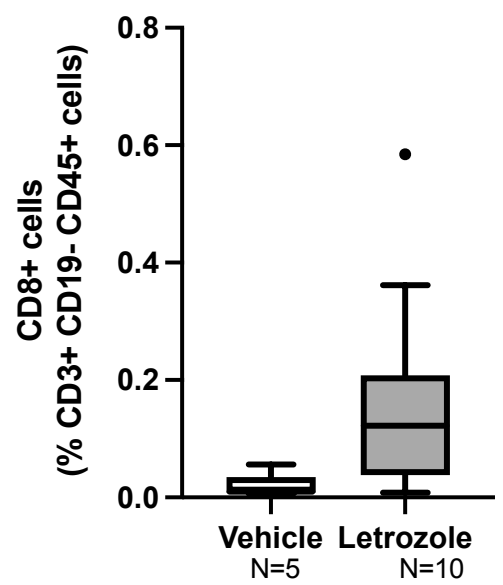**C**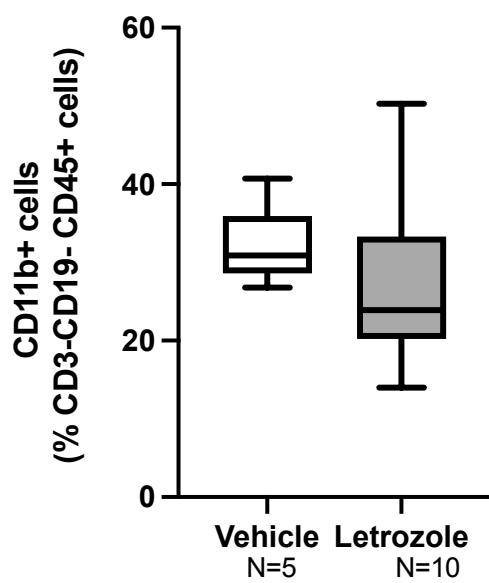**D**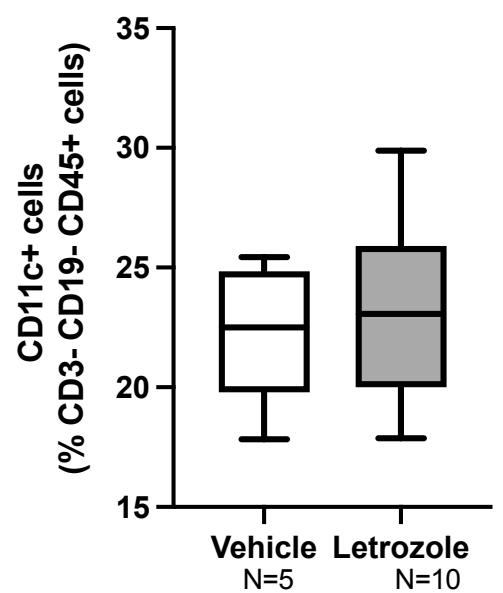**E**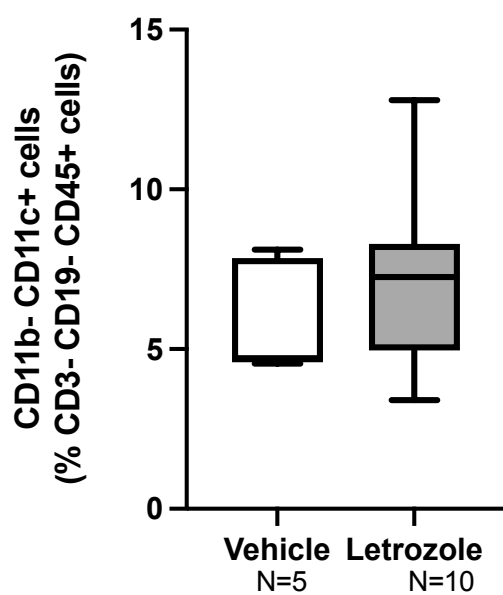**F**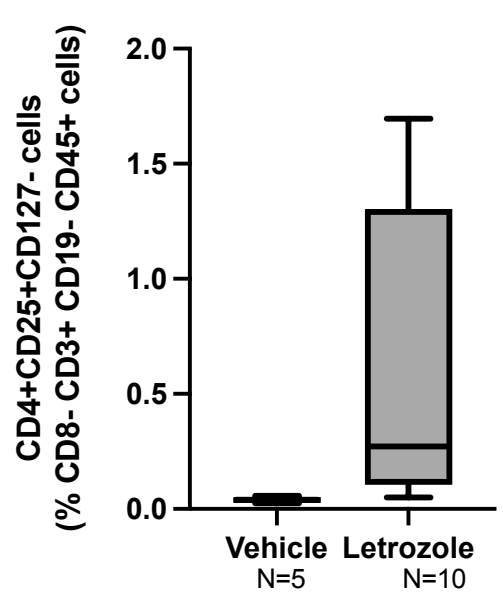

Supplement: Supplementary file 7 — Additional file 7. Analysis of remaining immune cell types in SSM3 Mouse tumours as determined by Flow Cytometry. (A) % CD45+ cells, (B) % CD8+, (C) % of CD11b+, (D) % of CD11c+, (E) % of CD11b-/CD11c+ and (F) % of CD4+ CD25+ CD127- cells. Error bars are the mean and SEM, and all cell types were non-significant as determined by unpaired t-test. [file 13058_2021_1472_MOESM7_ESM.pdf]
